# Ice-cover is the principal driver of ecological change in High Arctic lakes and ponds

**DOI:** 10.1371/journal.pone.0172989

**Published:** 2017-03-15

**Authors:** Katherine Griffiths, Neal Michelutti, Madeline Sugar, Marianne S. V. Douglas, John P. Smol

**Affiliations:** Paleoecological Environmental Assessment and Research Laboratory (PEARL), Department of Biology, Queen’s University, Kingston, Ontario, Canada; Institute of Tibetan Plateau Research Chinese Academy of Sciences, CHINA

## Abstract

Recent climate change has been especially pronounced in the High Arctic, however, the responses of aquatic biota, such as diatoms, can be modified by site-specific environmental characteristics. To assess if climate-mediated ice cover changes affect the diatom response to climate, we used paleolimnological techniques to examine shifts in diatom assemblages from ten High Arctic lakes and ponds from Ellesmere Island and nearby Pim Island (Nunavut, Canada). The sites were divided *a priori* into four groups (“warm”, “cool”, “cold”, and “oasis”) based on local elevation and microclimatic differences that result in differing lengths of the ice-free season, as well as about three decades of personal observations. We characterized the species changes as a shift from Condition 1 (i.e. a generally low diversity, predominantly epipelic and epilithic diatom assemblage) to Condition 2 (i.e. a typically more diverse and ecologically complex assemblage with an increasing proportion of epiphytic species). This shift from Condition 1 to Condition 2 was a consistent pattern recorded across the sites that experienced a change in ice cover with warming. The “warm” sites are amongst the first to lose their ice covers in summer and recorded the earliest and highest magnitude changes. The “cool” sites also exhibited a shift from Condition 1 to Condition 2, but, as predicted, the timing of the response lagged the “warm” sites. Meanwhile some of the “cold” sites, which until recently still retained an ice raft in summer, only exhibited this shift in the upper-most sediments. The warmer “oasis” ponds likely supported aquatic vegetation throughout their records. Consequently, the diatoms of the “oasis” sites were characterized as high-diversity, Condition 2 assemblages throughout the record. Our results support the hypothesis that the length of the ice-free season is the principal driver of diatom assemblage responses to climate in the High Arctic, largely driven by the establishment of new aquatic habitats, resulting in increased diversity and the emergence of novel growth forms and epiphytic species.

## Introduction

High-latitude environments have been identified as extremely vulnerable to climate change and are experiencing warming at about twice the rate of the global average [1]. In regions, such as the High Arctic, where monitoring records are either non-existent or are of short duration, the biological and chemical signatures of past environments preserved in the sediments of lakes and ponds (ubiquitous features in the Arctic landscape) can provide a long-term perspective on climate change. High-latitude waterbodies are commonly used for paleoclimate reconstructions [2] as they have demonstrated particular sensitivity to changes in climate [2, 3], with some lakes and ponds recording high taxonomic turnover in algal (e.g.[4–7]) and invertebrate assemblages (e.g. [8]). Furthermore, the remote nature of these sites means that they are often removed from direct anthropogenic disturbances, and so biological changes in these systems are largely driven by alterations in climate-associated processes. Sediment records from across the circumpolar Arctic have shown earlier and more dramatic biotic responses at the most northern sites, suggesting an increased sensitivity to anthropogenic climate change at higher latitudes [5].

The effect of climate warming on aquatic biota is not always straightforward, but is often predictable [2, 6, 7, 9]. In addition to increases in direct radiative heating, warming may lead to reductions in ice cover, resulting in longer growing seasons, the opening of new habitats for colonization, and the development of novel algal communities [4, 10]. Additionally, there may be associated changes in effective moisture (precipitation–evaporation) [3], higher rates of erosion, the development of thermal stratification, changes in nutrient concentrations and cycling, and greater catchment productivity, all of which may influence aquatic species [11]. A common algal group found in Arctic freshwater ecosystems is the diatoms (Bacillariophyceae) [11], siliceous algae that are ubiquitous and well-preserved in sediments [12]. Diatoms typically record indirect responses to warming such as to changes in habitat availability as the extent of ice cover diminishes [7, 13, 14], as well as to climate-associated changes in water chemistry (e.g. [15, 16]).

Early paleoecological work at Rock Basin Lake (unofficial name), east-central Ellesmere Island (Nunavut, Canada), suggested that ice cover was a dominant driver affecting the composition of diatom assemblages in northern environments [17]. The extensive summer ice and snow cover, it was argued, precluded the development of large populations of planktonic taxa, while the littoral assemblages were primarily driven by the fluctuation and extent of ice-free conditions in the littoral zone [10, 17]. Subsequent work on Arctic ponds at Cape Herschel (Ellesmere Island, Nunavut, Canada) identified increases in diatom diversity beginning in the mid-19th century, demonstrating the responsiveness of these ponds to climate changes, where slight temperature increases induced a marked change towards a more diverse and complex periphytic assemblage [4]. The shift in these Arctic ponds towards a diverse periphytic assemblage ([4], reviewed in [2, 18]) can be linked to an increase in the availability of exploitable epiphytic (living attached to vegetation) habitat, modified by the length of the ice-free season. Sites that develop vegetated substrates (often mosses at these latitudes), perhaps due to an extended ice-free season, are expected to experience a shift in their algal communities, from predominantly epilithic (living on rocks) and epipelic (living on sediment) species to greater proportions of epiphytic (living attached to vegetation) species, increasing the assemblage diversity [4, 5, 18].

Large High Arctic lakes can also undergo substantial ecological transformations, such as in typically perennially ice covered lakes on Ellesmere Island, where the loss of ice resulted in marked increases in algal production as, prior to warming, the extended ice cover greatly precluded diatom production [19–22]. To investigate the effect of ice-cover versus other limnological variables, Keatley *et al*. [23] examined diatoms in lake sediment records from two hydrologically connected lakes on northern Ellesmere Island that had different ice cover regimes. The lake that retained a raft of ice through the summer showed little change in contrast to the lake that was ice-free, which experienced dramatic shifts towards more diverse assemblages, consistent with the development of novel habitats as driven by ice-cover dynamics [23].

**Fig 1:**
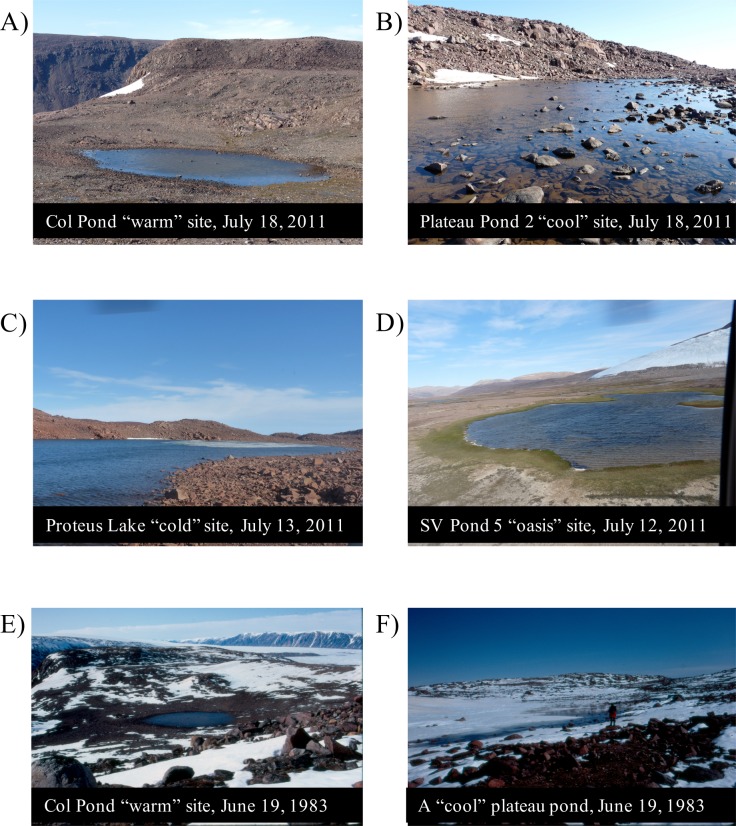
Images of the Ellesmere Island and Pim Island sites highlighting the differences in microclimate between the four categories. Col Pond (A) a “warm” site; Plateau Pond 2 (B) a “cool” site with snow persisting in the catchment; Proteus Lake (C) a “cold” site with no catchment vegetation and a pan of ice; and Sverdrup Pond 5 (D) an “oasis” site with considerable catchment vegetation. Photos A-D are taken from July 12^th^– 18^th^, 2011. Photo E and F are archival photographs (source John Smol) taken on June 19^th^, 1983, within one hour of each other. Col Pond (E) is ice free, while a pond on the plateau (F) remains largely ice covered (Plateau Pond 2, located ~0.5 km away and at a similar elevation, was fully ice-covered at the time), illustrating the differences in ice-off times between the “warm” and “cool” sites at the beginning of summer.

Here, we explicitly examine the role of ice cover as the dominant driver of diatom assemblage change by analyzing diatoms and aquatic production (as inferred from sediment chlorophyll *a* concentrations) in sediment records from four categories of lakes and ponds on east-central Ellesmere Island and Pim Island (Nunavut). The sites have been studied since 1983 as part of a long-term monitoring program (e.g. [2]) and represent four different local climate/ice cover regimes (Fig 1), with the site divisions based on the observations made over 30 years of site visits (see Table 1 for grouping rationale). The four categories are: 1) “warm” sites that currently have long ice-free periods and are known to have responded sensitively and early to anthropogenic climate change [4]; 2) “cool” sites that have shorter ice-free periods than the warm sites; 3) “cold” sites, where the waterbodies rarely lose their full ice cover; and 4) “oasis” sites that historically had elongated ice-free periods in summer, prior to anthropogenic warming.

**Table 1:** Rationale for the group divisions into “warm”, “cool”, “cold”, and “oasis” sites for the ten study sites from Ellesmere Island and Pim Island, Nunavut.

Site Group	Lake Name	Latitude	Longitude	Elevation (m a.s.l.)	Years sampled	Group division rational
“Warm” Sites	Col Pond	78° 36.154’ N	74° 39.758’ W	137	1983, 1984, 1986, 1987, 1995, 1998, 2001, 2004, 2006, 2007, 2009, 2011	Col Pond has never been ice covered during July sampling (at the height of summer) and the catchment has largely been free of snow. Col Pond was reported in 1984 as the first pond on Cape Herschel to lose its ice cover in early summer. Its location within a topographic col makes it one of the warmest sites on Cape Herschel.
	Elison Lake	78° 36.487’ N	74° 44.414’ W	23	1983, 1984, 1986, 1987, 1995, 1998, 2001, 2004, 2006, 2007, 2009, 2011	Elison Lake has rarely had ice cover or significant snow in the catchment during the July sampling visits. When the site was visited during our first three early season sampling events in June (1983, 1984, 1986), Elison Lake lost its ice cover around the same time as the “cool” sites (Moraine Pond and Plateau Pond 2). Elison Lake, because of its relatively larger size, is the last on Cape Herschel to freeze in late August. The low elevation and location on a valley floor are likely reasons why Elison Lake has one of the longest ice-free seasons on Cape Herschel.
“Cool” Sites	Moraine Pond	78° 36.685’ N	74° 40.977’ W	89	1983, 1986, 1987, 1995, 1998, 2001, 2004, 2006, 2007, 2009, 2011	Moraine Pond has had a persistent snowbank in its catchment on every sampling occasion, except near the end of the warm 2011 season. Additionally, Moraine Pond has occasionally been partially ice covered even during July sampling. Shading by the Cape Hershel cliffs maintains the snowbank on the north side of Moraine Pond and keep this site cool.
	Paradise Pond	78° 36.530’ N	74° 46.117’ W	134	1986, 1987, 1995, 1998, 2001, 2004, 2006, 2007, 2009, 2011	Paradise Pond is a relatively exposed site and at a high elevation, although Paradise Pond has never had ice cover during sampling (however it has always been sampled later in the season, in the middle of summer, as it is difficult to climb the cliff and access the site when snow and ice persist). It is fed partly by a large snowpack and regularly has other snowbanks in the catchment.
	Plateau Pond 2	78° 35.500’ N	74° 38.427’ W	246	1983, 1984, 1986, 1987, 1995, 1998, 2001, 2004, 2006, 2007, 2009, 2011	Plateau Pond 2 has had snow in its catchment on every sampling occasion. Additionally, Plateau Pond 2 has occasionally been partially ice covered even during our July sampling. Plateau Pond 2 is one of the highest elevation sites on Cape Herschel.
“Cold” Sites	High Lake	78° 42.700’ N	74° 22.283’ W	463	2011	The Pim Island lakes are high elevation, exposed sites. High Lake was not accessible to sampling due to extensive snow and ice cover until the warmer 2011 sampling season when it was completely ice-free. High Lake is our highest elevation site.
	Proteus Lake	78° 41.876’ N	74° 23.022’ W	376	1983, 1987, 1998, 2007, 2009, 2011	Proteus Lake consistently has been partially ice covered on every sampling occasion.
	West Lake	78° 44.491’ N	74° 37.751’ W	323	1983, 1987, 2009, 2011	West Lake has had extensive (90–100%) ice cover at each sampling occasion, except in July 2011 when it was completely ice free. West lake sits at a high elevation and is exposed.
“Oasis” Sites	Sverdrup Pond 5	79° 7.951’ N	79° 48.582’ W	299	2011	Sverdrup Pass is recognized as a “polar oasis” by ecologists. Although Sverdrup Pond 5 was only visited in 2011, other ponds within this polar oasis were visited in 1984 and the region was noted to host markedly more vegetation than Cape Herschel and Pim Island. Sverdrup Pass has been ice-free for ~5000–6000 years and was used as a migratory route by early peoples [24]. The perimeter of SV Pond 5 was entirely vegetated at the time of sampling in 2011.
	Sverdrup Pond 8	79° 7.680’ N	79° 58.498’ W	296	1984, 2011	Sverdrup Pass Pond 8 is located within Sverdrup Pass and in 1984 and 2011, the site was ice-free, lacked snow in the catchment, and hosted a 100% vegetated perimeter.

Rationale for group divisions is based on nearly 30 years of sampling and observations (1983–2011), with additional field note comments provided in S1 Table.

Given that a gradient exists in the length of ice cover between our four site groups (based on regular observations since 1983; Table 1), partly as a function of elevation and local microclimate (e.g. shaded or in sheltered valleys), we hypothesized that the greatest and earliest diatom assemblage shifts will be documented in the “warm” sites. The second largest changes are likely to be recorded in the “cool” sites that may have had delayed reductions in ice cover as compared to the “warm” sites, due to their higher elevation and/or local microclimatic conditions (Table 1). The “cold” sites, which typically still retain extended ice covers in summer, should only record muted and delayed changes in diatom assemblages. Additionally, we hypothesized that ponds located in the historically warm “oasis” Sverdrup Pass would not record any dramatic taxonomic turnover as, prior to recent anthropogenic warming, these sites already would have had relatively long ice-free periods and well-established aquatic vegetation, and therefore would have already supported diverse habitats for diatom growth. However, the diatoms at the “oasis” sites would likely reflect other climate-related limnological changes, such as increased summer production or decreases in the moisture balance due to enhanced evaporation.

## Site description

**Fig 2:**
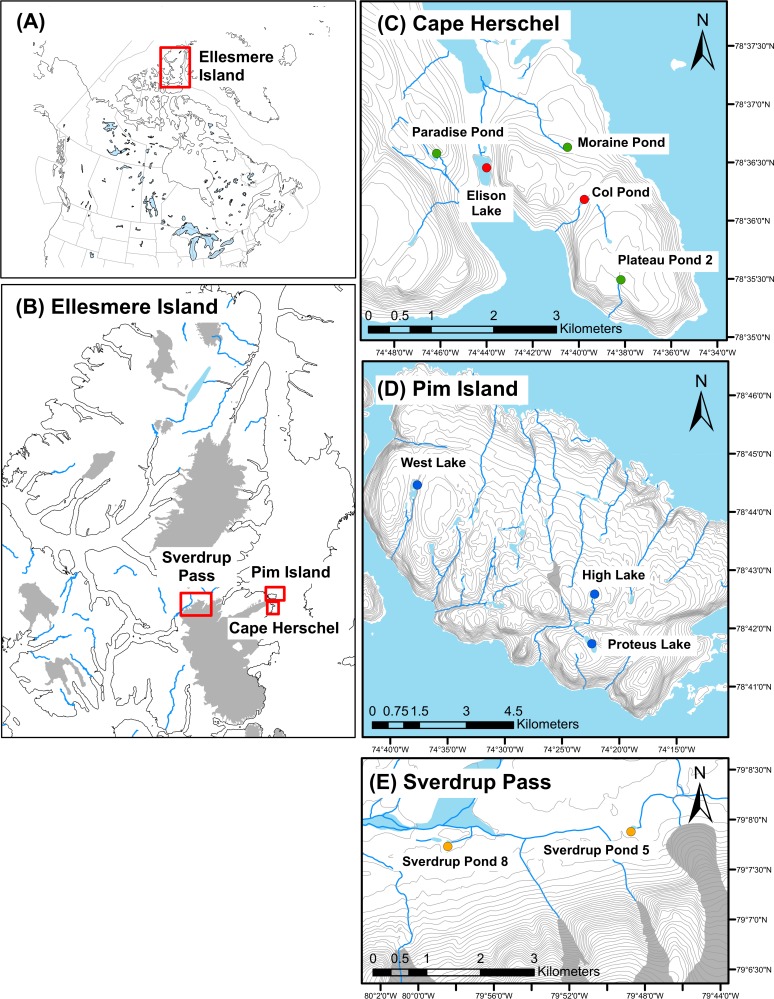
Location map of study sites. Location map for Ellesmere Island, Nunavut, Canada (A), with Ellesmere Island in detail highlighting the three study regions: Cape Herschel, Pim Island, and Sverdrup Pass (B), and higher resolution insets of Cape Herschel (C), Pim Island (D), and Sverdrup Pass (E) identifying the 10 lakes and ponds in this study with “warm” sites as red circles, “cool” sites as green circles, “cold” sites as dark blue circles, and “oasis” sites in orange. Major hydrological features are shown in light blue, glaciers in dark grey and 20 m topographic contours as light grey lines. Source: Natural Resources Canada [28].

We sampled ten lakes and ponds located on Cape Herschel (78° 37’N, 74° 42’W), east-central Ellesmere Island and on nearby (~10 km to the northeast) Pim Island (78° 43’N; 74° 27’W), and at Sverdrup Pass (79° 8’N, 79° 50’W), central Ellesmere Island (about 120 km from Cape Herschel), between July 12^th^ and 24^th^, 2011 (Fig 2). Select limnological and water chemistry variables for the ten sites are provided in Table 2. The Cape Herschel sites have been visited at irregular intervals since 1983 (Table 1) [25], as part of our long-term limnological monitoring program. Ponds (defined as waterbodies < 2 m deep) freeze to the bottom in winter.

**Table 2:** Coordinates, elevations and estimated maximum depths, and July 2011 water chemistry data for the ten study sites from Ellesmere and Pim Islands (Nunavut, Canada).

Site Group	Lake Name	Latitude	Longitude	Elevation (m a.s.l.)	Depth (m)	pH	Specific conductance (μS/cm)	DOC (mg/L)	DIC (mg/L)	TP_u_ (μg/L)	TN_f_ (mg/L)
“Warm” Sites	Col Pond*	78° 36.154’ N	74° 39.758’ W	137	0.5	8.2	143	1.9	13.4	6.4	0.193
	Elison Lake	78° 36.487’ N	74° 44.414’ W	23	1.5	8.4	357	4.0	19.3	6.0	0.365
“Cool” Sites	Moraine Pond*	78° 36.685’ N	74° 40.977’ W	89	0.5	8.8	208	2.3	19.6	13.6	0.243
	Paradise Pond*	78° 36.530’ N	74° 46.117’ W	134	1.8	8.4	35	0.8	1.4	4.4	0.118
	Plateau Pond 2*	78° 35.500’ N	74° 38.427’ W	246	0.3	8.1	74	2.0	4.3	6.6	0.199
“Cold” Sites	High Lake*	78° 42.700’ N	74° 22.283’ W	463	4.0	8.2	64	0.5	4.7	6.6	0.871
	Proteus Lake	78° 41.876’ N	74° 23.022’ W	376	6.9	8.7	73	1.0	3.4	4.2	0.114
	West Lake*	78° 44.491’ N	74° 37.751’ W	323	12.1	8.0	48	0.6	3.1	5.8	1.32
“Oasis” Sites	Sverdrup Pond 5*	79° 7.951’ N	79° 48.582’ W	299	0.5	8.6	365	14.6	37.8	15.8	1.11
	Sverdrup Pond 8*	79° 7.680' N	79° 58.498' W	296	0.3	8.7	571	20.1	51.5	13.9	6.56

Sites grouped according to local climate and/or ice cover regime where “warm” sites have relatively long ice-free periods, “cool” sites have shorter ice-free periods than the warm sites, “cold” sites rarely lose their full ice cover, and “oasis” sites historically had elongated ice-free periods in summer, prior to anthropogenic warming. The physical and chemical parameters include the estimated maximum depth (depth), dissolved organic carbon (DOC), dissolved inorganic carbon (DIC), total phosphorus (unfiltered) (TP_u_), and total nitrogen (filtered) (TN_f_) concentrations.

*Unofficial name

The climate of central Ellesmere Island is cold and dry, with mean annual temperature of -19°C, mean annual precipitation of 79 mm, and mean maximum daytime temperatures in the summer months (June, July, and August) of 6°C, 9°C, and 5°C, respectively [26]. The number of ice-free days is variable based on local climate conditions and elevation, typically ranging from 40 days to 65+ days [27]. We classified the ten sites into four groups based on local climate and ice cover regimes: 1) the “warm” sites; 2) the “cool” sites; 3) the “cold” sites; and 4) the “oasis” sites (Fig 2), as detailed in Table 1 and described more fully below.

### Group 1: The “warm” sites

The “warm” sites are located on Cape Herschel (Fig 2C), a cape comprised of Paleoproterozoic orthopyroxine granite with localized metasedimentary units [29, 30] [30] and with patchily distributed outcrops of calcareous glacial till [29]. Our research team has regularly visited and sampled the ponds on Cape Herschel, approximately every three years, for about the past 30 years. Indeed, Elison Lake and Col Pond on Cape Herschel were first analyzed for diatoms by Douglas *et al*. [4] using sediment cores taken in 1978 [31]. We re-cored these sites in 2011 to capture more recent (post-1978) changes in their sedimentary records. The two “warm” ponds (depth < 2 m), Col Pond and Elison Lake, are thought to have the longest ice-free seasons on Cape Herschel (Table 1 and S1 Table). Col Pond is located within a topographic col at a moderately high elevation for Cape Herschel (137 m a.s.l.). Located at the bottom of a valley and protected by the wind in two directions, Col Pond is amongst the first of the ponds on Cape Herschel to lose its ice cover (Table 1, Fig 1E). Elison Lake, despite its name, is a large pond (209 m x 584 m, max depth 1.5 m) at low elevation (32 m a.s.l.). Its relative large size and low elevation make it one of the last on Cape Herschel to freeze at the end of the short summer (typically mid- to late August). A thermistor installed at a depth of 0.5 m in Elison Lake recorded an average of 92 ice-free days/year (from 2008–2010, determined as the number of days in one year between pond temperatures rising above 0°C to when they first dip below 0°C). Both ponds are relatively shallow, slightly alkaline, and oligotrophic (Table 2), and both contained submerged aquatic mosses at the time of sampling.

### Group 2: The “cool” sites

The “cool” sites are also located on Cape Herschel (Fig 2C) and are amongst the last on Cape Herschel to lose their ice cover, as these ponds have little shelter and are at relatively high elevations (Table 1). The three “cool” ponds (< 2 m deep) are Moraine Pond, Paradise Pond, and Plateau Pond 2. Moraine Pond is shaded by cliffs and is fed by a stream that drains one of the largest catchments (including the large cliffs to the south) for the Cape Hershel ponds and is mesotrophic (Table 2), while Paradise Pond and Plateau Pond 2 were oligotrophic at the time of sampling (Table 2). All ponds were slightly alkaline at the time of sampling in July 2011. On previous sampling occasions, however, the pH at Paradise Pond has generally been lower (average pH over all sampling occasions 7.2 ± 0.6, n = 10; additionally, this is the only site on Cape Herschel where the pH has been measured below 7), and has the lowest DIC of all our sites (Table 2), reflecting the pond’s relatively poor buffering capacity. All the “cool” ponds had persistent snow banks in the catchment when sampled in 2011. Submerged aquatic mosses were observed in Moraine Pond at the time of sampling, while only wet shoreline mosses located between boulders were present in Plateau Pond 2. Paradise Pond did not have any aquatic mosses for the July 2011 sampling.

### Group 3: The “cold” sites

Pim Island is a steep-sided island located ~10 km to the north of Cape Herschel and composed of Paleoproterozoic orthopyroxine granite with metasedimentary enclaves [30], with occurrences of discontinuous calcareous till [32]. The exposed, high elevation ponds on the island had no appreciable accumulation of sediment for coring, and therefore we focused on obtaining sediment cores from deeper lakes. The “cold” Pim Island sites are: High Lake, Proteus Lake, and West Lake (Fig 2D). Our research group sampled these sites for 1, 6, and 4 summers, respectively, over the past 30 years. However, in addition to direct field sampling, we have completed a number of helicopter surveys noting ice covers in other years. Typically, these high elevation lakes have between 90 and 100% ice cover at the time of sampling, often even at the height of summer in late July and early August (Table 1), with summer melt limited to a narrow moat along the shore. However, during sampling in 2011, High Lake and West Lake were ice-free, while Proteus Lake was largely ice-free. The lakes were dilute, oligotrophic and slightly alkaline at the time of sampling in July 2011 (Table 2). No aquatic vegetation was observed at the “cold” sites at the time of sampling. Catchment vegetation for the Pim Island sites was routinely noted as being barren or sparsely vegetated.

### Group 4: The “oasis” sites

The two “oasis” sites are located in Sverdrup Pass, central Ellesmere Island (Fig 2E). Sverdrup Pass has been called a “polar oasis” due to its warmer temperatures and atypically greater biological production and diversity relative to ecosystems at similar latitudes [33]. The growing season here spans from late May to early September [34], and the warmer conditions have resulted in greater vegetation growth as compared to polar desert sites, with enough wet sedge meadow habitat to support a small muskox population [35]. The pass is an ice-free, U-shaped valley bordered by high cliffs of Cambrian and Lower Ordovician carbonates [36] and the study ponds are located within a variably argillaceous and arenaceous Cambrian limestone-dolostone [30]. The protection from wind by the cliffs (e.g. [37]) in combination with the low albedo, which may be amplified due to the parabolic nature of the valley, might explain the relatively warmer local climate in the Sverdrup Pass valley. Our two “oasis” sites are Sverdrup Pond 5 and Sverdrup Pond 8. The ponds had abundant lichen, bryophytes and vascular plants (predominantly cotton grass, *Eriophorum callitrix*) in their catchments, and 100% vegetated perimeters, characterizing them as notably more productive than the polar desert regions of Cape Hershel and Pim Island (Table 1). Both ponds were mesotrophic (Table 2) and had abundant submerged macrophytes at the time of sampling.

## Methods and materials

### Sediment collection and dating

The Arctic research was conducted under the guidelines outlined in J. Smol’s Nunavut Scientific Research License, issued by the Nunavut Research Institute (Iqaluit, Nunavut, Canada). Sediment cores were extracted from the near the deepest part of the lakes and ponds using either a Glew gravity corer [38], or push corer for waters < 1 m deep [39] avoiding any areas with visible cryogenic disturbances. Sediments were extruded on site using a Glew extruder [40] at 0.25 cm or 0.5 cm intervals.

Col Pond and Elison Lake were first cored in 1978 and dated using ^14^C and ^210^Pb chronologies [4]. The cores we collected in 2011 were matched to the ^210^Pb-dated cores from 1978 [4] using the marked shifts in the diatom assemblages, thus bringing the diatom records to 2011. The sediment cores for the “cool”, “cold” and “oasis” sites were freeze-dried and dated (at 0.5 or 1 cm intervals) using ^210^Pb gamma spectrometry [41, 42] at the Paleoecological Environmental Assessment and Research Laboratory at Queen’s University, Kingston, ON, Canada, and activities were converted to dates by applying the Constant Rate of Supply (CRS) method [43].

The ^210^Pb activities in the SV Pond 5 core were too low to obtain reliable dates. In lieu of ^210^Pb dates, we obtained basal dates for SV Pond 5 and SV Pond 8 by assessing the ^14^C in terrestrially-derived material (woody herbaceous stem fragments (SV Pond 5 and SV Pond 8), sedge *Carex* sp. achenes (SV Pond 8) and partial leaves of *Dryas integrifolia* and *Cassiope* sp. (SV Pond 5)), isolated by Paleotec Services in Ottawa, Canada, with the AMS radiocarbon dating performed at the Keck Carbon Cycle AMS Laboratory at University of California Irvine. The ^14^C ages were calibrated to years before present (cal yr BP) with the IntCal13 northern hemisphere terrestrial ^14^C calibration curve [44].

### Sediment processing

The sediment intervals were processed for diatoms following standard techniques [45]. A minimum of 350 diatom valves per interval (following [46]) were identified to the species and, when possible, variety levels, following Krammer and Lange-Bertalot [47–50], Douglas and Smol [27], and Antoniades *et al*. [51]. Diatom counts were converted into species relative abundances. Primary production was inferred using from sediment chlorophyll *a* (hereafter chl *a*) concentrations measured using visible reflectance spectroscopy (VRS), a method demonstrated to faithfully track trends in primary production [52, 53].

### Data analysis

Relative abundance stratigraphic diagrams were produced using C2 version 1.7.4 [54]. Only species that occurred at >5% in two or more intervals were plotted, otherwise taxa were grouped by genera or as “other taxa”. Diatom taxa were ordered in the stratigraphic profiles according to habitat preference, defined by the periphytic substrate the taxon was most commonly associated with, or on which it was found at the highest abundances, according to Douglas *et al*. [27], Lim *et al*. [14], Michelutti *et al*. [55], Kingston [56], and Kociolek and Spaulding [57]. Stratigraphic zones were identified by applying constrained, incremental sum-of-squares cluster (CONISS) analyses [58] and the significance of the divisions was determined by a broken stick model [59] in the “vegan” package v. 2.0–10 [60] for the R software environment [61]. Prior to calculating species diversities, diatom species raw counts were rarefied to a common sum of 350 valves and the species diversity represented by Hill’s N2 [62], which takes into account both evenness and species richness, was calculated for each site. The CONISS, the broken stick, rarefaction, and Hill’s N2 analyses were completed using the “vegan” package in R. Due to differences in the morphometry and catchments of the waterbodies, resulting in marked differences in baseline production, the chl *a* values for each group of sites were plotted on relative scales. To highlight the timing of species changes, the significant CONISS breaks for each site are indicated in the figures.

## Results

### Dating

**Fig 3:**
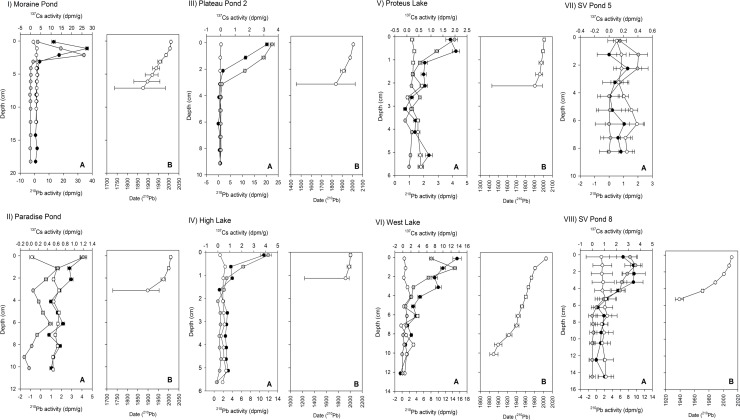
Profiles of ^210^Pb activities and age-depth models. The activities in decays per minute g^-1^ (dpm/g) of unsupported ^210^Pb (black circles), supported ^210^Pb (white circles) and ^137^Cs (grey circles) *vs* sediment depth (A) and the Constant Rate of Supply ^210^Pb generated age-depth model (B) for each dated study site: Moraine Pond (I), Paradise Pond (II), Plateau Pond 2 (III), High Lake (IV), Proteus Lake (V), West Lake (VI), SV Pond 5 (VII), and SV Pond 8 (VIII). Error bars represent standard error.

Unsurprisingly for High Arctic lakes and ponds [63], the ^210^Pb activities were low for our dated sediment cores (Fig 3A). Peaks in the ^137^Cs activity were identified in three of the dated ponds (Moraine Pond, Fig 3A-I; West Lake, Fig 3A-VI; and SV Pond 8, Fig 3A-VIII) and two of these (Moraine Pond and West Lake) were generally in good agreement with the ^210^Pb CRS dates. To compare diatom species, diversity, and chl *a* changes across the dated sites, we extrapolated our age models back to the base of the cores. Mentions of specific dates are only meant to orient the reader to specific diatom, chl *a*, or diversity changes within the sediment profiles. We acknowledge the limitations of our dating models and, rather than specific inferences, we have limited the interpretation of our dates to broad characterizations. Generally, unsupported ^210^Pb activities decay to background levels (supported ^210^Pb activities) after ~150 years [42, 63]; however, this basal date can be younger in areas of low deposition where the difference between supported and unsupported ^210^Pb may not be possible to distinguish past 3–4 half-lives (60–90 years) due to analytical limits of detection [42]. Generally, where diatom changes occur in regions of the core where there is detectable unsupported ^210^Pb activity (such as Moraine Pond, Paradise Pond, High Lake, and Proteus Lake), we interpret these changes as having occurred later than assemblage shifts occurring in sediments where the unsupported ^210^Pb is depleted or the where changes occur at ~1850 AD (Col Pond and Elison Lake) based on matching diatom changes to a previously published dated profile [4]. The ^210^Pb activities in SV Pond 5 were too low to develop a dating model (Fig 3A-VII) and so radiocarbon basal dates were obtained for both SV Pond 5 and SV Pond 8, which dated the base of the cores to ~2265 ± 81 (UCIAMS # 141781) calibrated years before present (cal. yr BP) (at a depth of 9.5–10 cm) for the Sverdrup Pond 5 record and ~3064 ± 79 (UCIAMS # 121781) cal. yr BP (at a depth of 10–11 cm) for the Sverdrup Pond 8 record.

### Diatom assemblage changes

**Fig 4:**
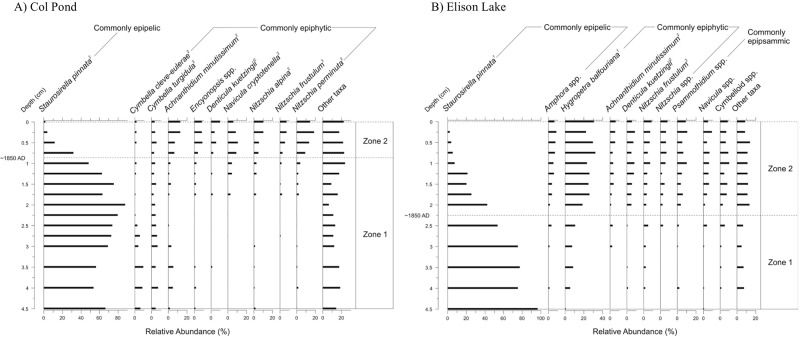
Relative frequency profiles of dominant diatom taxa sorted according to habitat preference from “warm” sites, along with significant stratigraphic zones. (A) Col Pond and (B) Elison Lake, on Ellesmere Island, Nunavut, Canada. Habitat preferences are provided and attributed as follows: 1) Douglas *et al*. [27], 2) Lim *et al*. [14], 3) Michelutti *et al*. [55], 4) Kingston [56], and 5) Kociolek and Spaulding [57]. Stratigraphies are plotted against core depth and reference dates are provided from a previous study [4] on the same sites.

The “warm” sites exhibit striking diatom changes (Col Pond: Fig 4A and Elison Lake: Fig 4B) near the mid- to late nineteenth century (estimated here to be ~1850 AD based on matching species changes to a previously dated sediment core [4]) with a shift from assemblages dominated by the epipelic taxon *Staurosirella pinnata* (CONISS zone 1) to more diverse assemblages including the emergence of epiphytic taxa (e.g. *Hygropetra balfouriana*) and *Nitzschia frustulum*, *N*. *alpina* and *N*. *perminuta* (CONISS zone 2).

**Fig 5:**
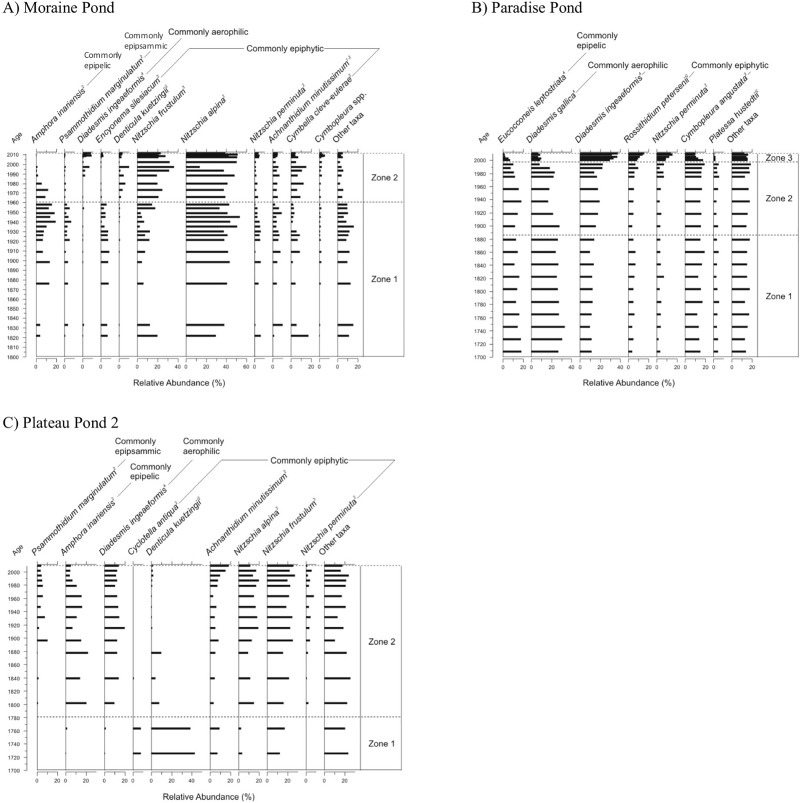
Relative frequency profiles of dominant diatom taxa sorted according to habitat preference from “cool” sites, along with significant stratigraphic zones. (A) Moraine Pond, (B) Paradise Pond, and (C) Plateau Pond 2, on Ellesmere Island, Nunavut, Canada. Habitat preferences are provided and attributed as follows: 1) Douglas *et al*. [27], 2) Lim *et al*. [14], 3) Michelutti *et al*. [55], 4) Kingston [56], and 5) Kociolek and Spaulding [57]. Stratigraphies are plotted against interpolated ^210^Pb age.

The diatom species shifts at the “cool” sites are not seemingly synchronous between ponds, but are nonetheless evident in the records. At Moraine Pond, the principal shift, marked by the transition from CONISS zone 1 to 2, is an increase in *Nitzschia frustulum* around 1960 AD, and a concomitant decrease in the relative abundance of the epipelic *Amphora inariensis* (Fig 5A). At Paradise Pond, the first significant assemblage change (CONISS zone 1 to 2) occurs ~1880 AD and is characterized by a slight relative increase in the aerophilic (found at exposed sites) *Diadesmis ingeaeformis* at the expense of another aerophilic taxon, *Diadesmis gallica* (Fig 5B). The second significant transition (CONISS zone 2 to 3) occurs at between ~1994 AD and ~2000 AD, with a similar decrease in *D*. *gallica* and an increase in *D*. *ingeaeformis*, but accompanied by increases in *Nitzschia perminuta* and epiphytic *Rossithidium petersenii*, and a decrease in the epipelic *Eucocconeis leptostriata*.

In Plateau Pond 2, we observed that the diatom valves preserved in the sediment pre-1800 AD are mostly broken. The transition from CONISS zone 1 to 2 in the diatom assemblage occurs at an extrapolated age of ~1780 AD, marking a change from a *Denticula kuetzingii* and *Cyclotella antiqua*-dominated assemblage, to a more diverse assemblage of *Amphora inariensis*, *Nitzschia* spp. and *Diadesmis ingeaeformis* (Fig 5C). There are no other significant zones for this site; however, there is a non-significant change in the assemblage around 1880 AD, marked by a decrease in the epipelic *Amphora inariensis*, and increases in *Nitzschia perminuta*, *Psammothidium marginulatum* and *Achnanthidium minutissimum*. In the most recent sediments, there is a decline in the relative abundance of *Amphora inariensis* at ~ 1980 AD, with a corresponding increase in *Achnanthidium minutissimum*.

**Fig 6:**
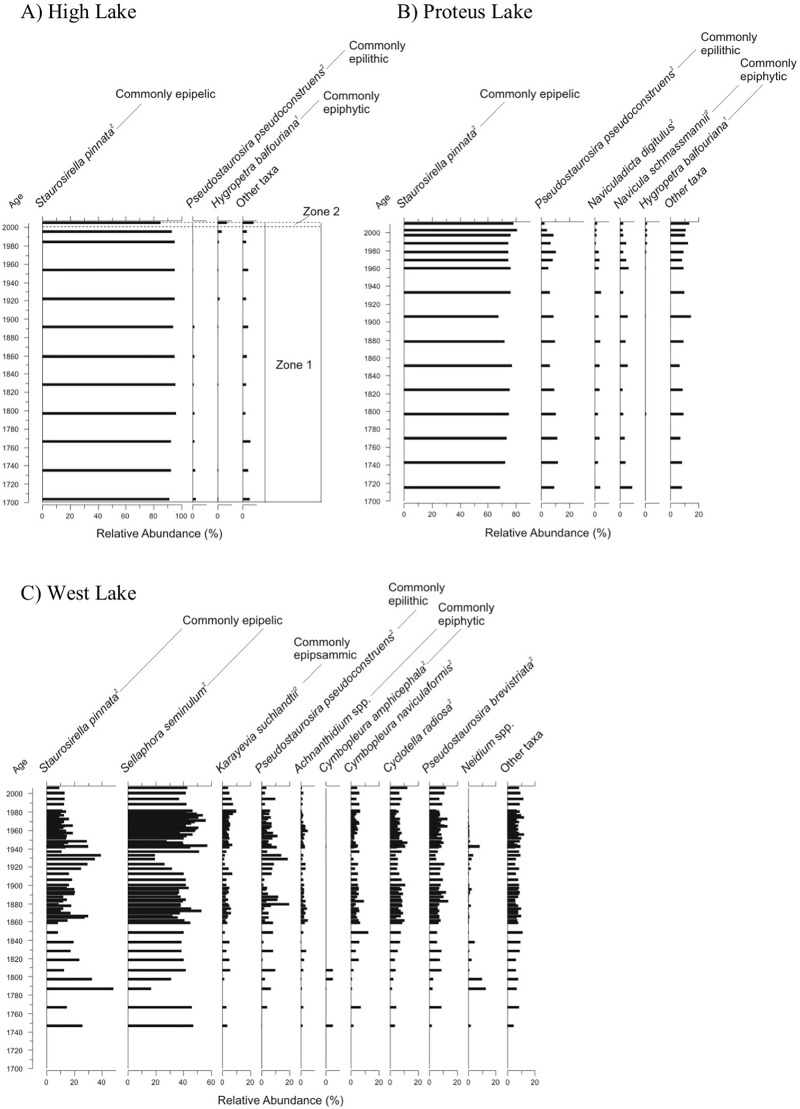
Relative frequency profiles of dominant diatom taxa sorted according to habitat preference from “cold” sites, along with significant stratigraphic zones. (A) High Lake, (B) Proteus Lake, and (C) West Lake, on Pim Island, Nunavut, Canada. Habitat preferences are provided and attributed as follows: 1) Douglas *et al*. [27], 2) Lim *et al*. [14], 3) Michelutti *et al*. [55], 4) Kingston [56], and 5) Kociolek and Spaulding [57]. Stratigraphies are plotted against interpolated ^210^Pb age.

Diatom assemblages at the “cold” sites show little to no directional change in their records. High Lake (Fig 6A) and Proteus Lake (Fig 6B) have assemblages dominated by the small, benthic, epipelic and epilithic fragilarioid taxa (e.g. *Staurosirella pinnata*, *Pseudostaurosira pseudoconstruens*). The High Lake and Proteus Lake records show a slight increase in the epiphytic *Hygropetra balfouriana* in the transition from CONISS zone 1 to 2, although in Proteus Lake the relative abundances of this diatom are slightly below the cut-off criteria of >5% in two or more intervals, but this taxon is included in the stratigraphy due to its ecological significance (Fig 6B). The West Lake record similarly displays no directional assemblage change, although it is more variable than the other “cold” records (Fig 6C) with fluctuating dominance between two epipelic species, *Staurosirella pinnata* and *Sellaphora seminulum*.

**Fig 7:**
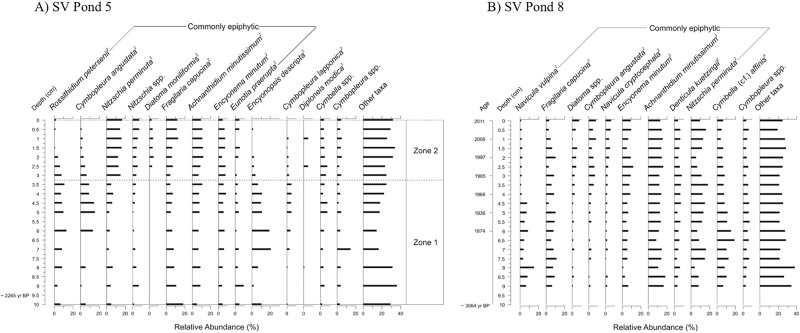
Relative frequency profiles of dominant diatom taxa sorted according to habitat preference from Sverdrup Pass “oasis” sites, along with significant stratigraphic zones. (A) SV Pond 5 and (B) SV Pond 8, Ellesmere Island, Nunavut, Canada. Habitat preferences are provided and attributed as follows: 1) Douglas *et al*. [27], 2) Lim *et al*. [14], 3) Michelutti *et al*. [55], 4) Kingston [56], and 5) Kociolek and Spaulding [57]. Stratigraphies are plotted against core depth and reference dates are provided from ^210^Pb ages, where suitable (B), and with basal ^14^C ages.

The “oasis” sites, SV Pond 5 and SV Pond 8, record more muted diatom species changes than the “warm” sites. In contrast to non-oasis sites, the composition of diatoms in the early sediments from these ponds consist of more complex assemblages that include a variety of epiphytic forms (Fig 7). At SV Pond 5, the principal CONISS shift from zone 1 to zone 2 is marked by a decrease in *Encyonopsis descripta*, *Rossithidium petersenii*, and *Cymbopleura angustata*, and an increase in the relative abundance of *Nitzschia perminuta* and an increase in *Diatoma moniliformis*, although at abundances slightly below the cut-off criterion (Fig 7A). SV Pond 8 had no significant directional change in the assemblage; however, there is a slight shift around 1920 AD with a decline in *Navicula vulpina* and *Cymbella* (cf.) *affinis* and an increase in *Diatoma* spp., *Cymbopleura angustata* and *Navicula cryptocephala* (Fig 7B).

### Hill’s N2

**Fig 8:**
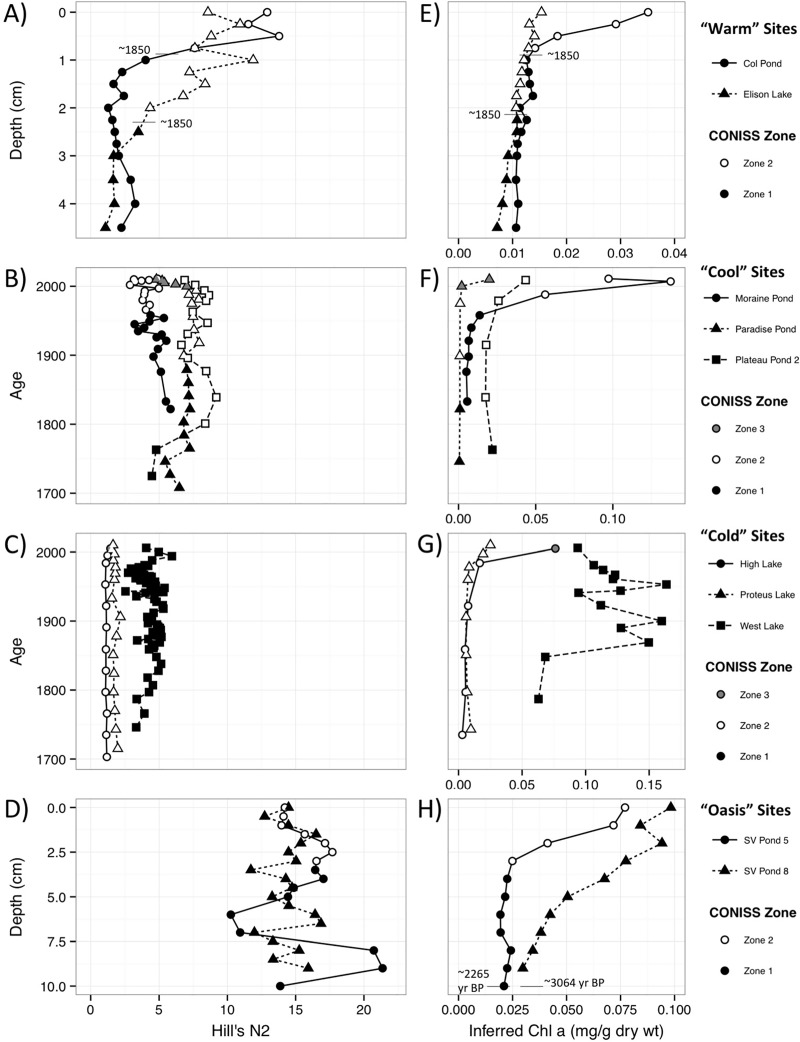
Hill’s N2 diversity index values chlorophyll *a* profiles for all study sites. Hill’s N2 diversity index values (A, B, C, D) and visible reflectance spectroscopy-inferred chlorophyll *a* concentrations (E, F, G, H) from lakes and ponds divided into “warm” (A, E), “cool” (B, F), “cold” (C, G), and “oasis” (D, H) groups based upon local elevation and climate gradients on Ellesmere Island and Pim Island, Nunavut, Canada. The diatom zones are indicated on each profile to relate the species changes to the changes in diversity and inferred primary production.

Diatom diversity, assessed by the effective number of “very abundant” species (Hill’s N2), increased markedly at the “warm” sites since the mid-19th century (Fig 8A). The significant CONISS zones mark the increases in Hill’s N2, with a transition from pre-1850 AD assemblages to higher diversity post-1850 AD assemblages. In contrast, the “cool”, “cold” and “oasis” sites have maintained relatively steady Hill’s N2 values (Fig 8B, 8C and 8D, respectively). The “cold” Pim Island lakes (Fig 8C), which retain large portions of ice cover though the summer months, have low effective numbers of species, similar in diversity to the pre-1850 AD sediment at the “warm” sites (average Hill’s N2 and standard deviation 2.6 ± 1.5). The “oasis” sites (Fig 8D), which were historically vegetated and ice free, have Hill’s N2 values similar to the post-1850 AD “warm” assemblages (average Hill’s N2 and standard deviation 14.9 ± 2.3).

The “cool” sites recorded an intermediate effective number of species between that of the “cold” sites and “oasis” sites (Fig 8B) (average Hill’s N2 and standard deviation 6.2 ± 1.6). Generally, the significant CONISS zones for the “cool” sites do not correspond with shifts in Hill’s N2. Rather, the CONISS zones for the “cool” sites reflect species transitions from largely epilithic and epipelic forms towards epiphytic species (see Fig 5). The timing of these assemblage shifts are variable, with Moraine Pond, Paradise Pond, and Plateau Pond 2 changing at ~1960 AD, ~1995 AD, and ~1880 AD respectively.

### Sediment chlorophyll *a*

The VRS sediment chlorophyll *a* method measures both primary chlorophyll *a* and its dominant diagenetic products, which provides a reliable overall of whole lake production trends [52, 53]. Profiles of our Arctic sites indicate that most of the lakes and ponds have experienced increased primary production over the past ~200 years. The “warm” site, Col Pond, displayed marked increases in chl *a* (Fig 8E), corresponding with an increase in Hill’s N2 (Fig 8A). The larger, deeper “warm” site, Elison Lake, demonstrated a much subtler change in primary production with low concentrations of chl *a* that nearly doubled over the record (Fig 8E), although the concentrations even in the uppermost sediment remain quite low. The slight increase in chl *a* is concurrent with the increase in Hill’s N2.

The chl *a* increases in the “cool” site profiles (Fig 8F) are generally consistent with the CONISS breaks in the diatom profiles, suggesting an increase in primary production contemporaneous with the relative increase in epiphytic diatom species. Sedimentary chl *a* has also increased in two of the three “cold” sites (High Lake and Proteus Lake), with West Lake displaying a high variability and little directionality in the chl *a* record (Fig 8G). For High and Proteus lakes, increases in chl *a* (Fig 8G) are coincident with subtle increases in *Hygropetra balfouriana*, an epiphytic taxon (Fig 6A and 6B).

In contrast to the Hill’s N2 data, which show little directional change (Fig 8D), the chl *a* profiles from the “oasis” sites reflect substantial increases in primary production (Fig 8H). The increase in chl *a* in SV Pond 5 coincides with increases in *Diatoma* spp. and decreases in *Cymbopleura angustata*, *Encyonopsis descripta*, and *Rossithidium petersenii* (Fig 7A). Chl *a* also increases in SV Pond 8, and while this increasing trend is evident prior to ~1870 AD, the increase is more marked after this date.

## Discussion

### Diatom assemblage shifts and changing aquatic conditions

The four categories of Arctic ponds that we identified as having differing lengths of the ice-free season, due to differences in elevation and microclimate, have recorded distinct responses in their diatom assemblages. Our sites can be divided into two conditions based on the diversity and the relative frequencies of diatoms indicative of longer growing seasons and often associated with aquatic vegetation [18]. The first condition (Condition 1) often has low diversity and the diatom assemblages are largely dominated by prostrate, non-motile, adnate benthic epipelic and epilithic taxa, suggesting a short growing season with little to no establishment of aquatic vegetation [18]. The second condition (Condition 2) is typically marked with higher diversity and the presence or dominance of motile epiphytic diatom taxa, often with complex growth forms (e.g. motile, stalked or tube dwelling), indicating a long growing season and the presence of aquatic vegetation.

The diatom records from the “cold” sites (Fig 6) and the “oasis” sites (Fig 7) exemplify Condition 1 and Condition 2, respectively. The “cold” sites have the lowest diversity of our four groups (Fig 8C), with assemblages dominated by the small, benthic fragilarioid taxa (Fig 6). The benthic fragilarioid taxa are opportunistic, pioneering species that are non-motile, typically epipelic and epilithic, and are characteristic of cold, alkaline, oligotrophic waters [6, 64, 65]. In Arctic regions, these taxa are often amongst the first to colonize lakes and ponds following deglaciation (e.g. [4, 66]) and ice off [20] as they are able to exploit these rather harsh environments that are characterized by extended ice cover, low light, and low nutrients, which might exclude other taxa [64, 65, 67, 68]. No aquatic vegetation was observed in the “cold” sites during sampling, and the scarcity of epiphytic taxa and historically low diversity suggest there has been little change to diatom growing conditions over most of the record. However, in the most recent period (after ~2000 AD), there are subtle indications that limnological conditions are shifting, particularly at High Lake and Proteus Lake (Fig 6A and 6B). Diatom assemblages in the most recent intervals from these sites have undergone minor increases in *Hygropetra balfouriana*, a motile moss epiphyte that has previously been identified as indicating warming [4], suggesting the lakes may be in the process of transitioning from Condition 1 (low diversity and/or epilithic/epipelic species) to Condition 2 (higher diversity and/or epiphytic species). These changes indicate that we might have captured the early stages of this threshold-like condition shift in our sediment record.

The diatom profile from West Lake shows no directional assemblage change, although it is more variable as compared to the other “cold” records (Fig 6C) with fluctuations between two epipelic species. The dominant species and high variability are consistent with the late Holocene assemblages from a 10,000 year diatom record of West Lake [69] suggesting the shifts in our short record are generally not outside the range of variability of the past 4000 years.

In contrast to the low diversity “cold” sites (Fig 8C), the two “oasis” sites from Sverdrup Pass had, as hypothesized, the highest diatom diversities (Fig 8D), but have remained generally stable throughout the sediment core. Our data suggest that the “oasis” ponds currently, and likely historically, have supported aquatic mosses and relatively lush catchments [70, 71] and, as a result, epiphytic species dominate the fossil diatom record (Fig 7) with taxa that resemble assemblages from other Arctic oasis sites [37]. The diatom changes recorded in the “oasis” ponds do not reflect novel habitat development, but rather changes, perhaps, between epiphytic habitat of different types (e.g. submerged grasses versus aquatic mosses) or shifts in water chemistry related to warming. For example, the increase in both “oasis” ponds of *Diatoma* taxa (including *D*. *moniliformis*, *D*. *tenuis*, and *D*. *vulgaris*), taxa often recorded in higher conductivity waters [72], may be due to increased evaporation due to the warming climate concentrating solutes in the pond, a trend that has been recorded in similarly shallow ponds across the Arctic [3].

The “warm” study sites (Fig 4) have recorded a marked transition in their diatom assemblages beginning relatively early (~1850 AD), shifting from low diversity (Fig 8A) and predominantly benthic taxa (Condition 1) (Fig 4) towards higher diversity and more complex epiphytic assemblages (Condition 2). Additionally, *Nitzschia* spp. (*N*. *frustulum*, *N*. *alpina* and *N*. *perminuta*) increase in relative abundances concurrent with the diversity shift at both “warm” sites. In the Arctic, these *Nitzschia* taxa are often found in waterbodies with higher nutrients [14, 73] or may bloom in response to an influx of nutrients, such as the input of sewage effluent into pristine Arctic waters [74, 75]. At our sites, the increase may reflect a greening of the catchment based upon local observations over the past ~30 years of field seasons and reflective of the general trend seen across the Canadian Arctic [76, 77], and the consequent delivery of additional nutrients to the typically ultra-oligotrophic Arctic ponds.

At the “cool” sites (Fig 8B), the diversity is slightly higher than for the “cold” sites (Fig 8C), but the Hill’s N2 diversity values show little directional change with time. However, in contrast to the “cold” sites (Fig 6), there are significant taxonomic changes in the diatoms assemblages in each of the “cool” records (Fig 5), reflecting the shift from mainly epipelic and epilithic taxa (Condition 1) to increasing numbers of epiphytes and species characteristic of higher nutrients (i.e. *N*. *frustulum*, *N*. *alpina* and *N*. *perminuta* [14, 73]) (Condition 2). The shifts in the diatom assemblages occur at ~1960 AD, ~1995 AD, and ~1880 AD at the three “cool” sites (Moraine Pond (Fig 5A), Paradise Pond (Fig 5B) and Plateau Pond 2 (Fig 5C), respectively). The diatom shifts in the Moraine Pond and Paradise Pond records occurred prior to the complete decay of unsupported ^210^Pb in the sedimentary record and, as such, these changes can be cautiously interpreted as occurring after the changes at the “warm” sites. The variable timing of the shift from Condition 1 to 2 at the “cool” sites is in contrast to the “warm” sites, which transitioned earlier (~1850 AD), suggesting that the “warm” sites were particularly sensitive to even small changes in climate [4].

At Moraine Pond, the change from Condition 1 to Condition 2 occurs ~1960 AD (Fig 5A) and is marked by a relative decrease in the abundance of epipelic *Amphora inariensis* and a concomitant increase in the commonly epiphytic and nutrient-associated *Nitzschia frustulum* [73, 74]. In the Paradise Pond record, there are two significant CONISS zones (Fig 5B), but the second (~1995 AD) is the more ecologically relevant as it reflects a change in the habitats available for exploitation, likely marking a change from a short growing season with little to no vegetation (Condition 1) to the development of a complex, vegetated substrate (Condition 2). The first CONISS zone occurs in ~1880 AD and is marked by slight shifts in the relative abundances of two aerophilic *Diadesmis* spp. However, the ecological affinities of these aerophilic taxa are not adequately delineated to allow for meaningful ecological interpretations. On the other hand, the second transition (~1995 AD) records similar shifts in *Diadesmis* spp., but these changes are accompanied by increases in epiphytic and nutrient-associated taxa and a relative decrease in epipelic species. This shift is consistent with the field observations, as aquatic mosses were first identified in 1995 AD in Paradise Pond, providing additional support that the sediment record is capturing the transition from Condition 1 to Condition 2 in the mid-1990s.

Plateau Pond 2 recorded an early shift in the diatom record around 1780 AD (extrapolated age), marking a change from a *Denticula kuetzingii* (a species that has been associated submerged mosses [55] and shoreline mosses [78]) and a benthic *Cyclotella antiqua*-dominated assemblage (a diatom found in the littoral of lakes and rivers; [79]), to a more diverse assemblage (Fig 5C). The high proportion of broken diatom valves in the pre-1800 AD sediments perhaps suggests that the diatoms may be largely allochthonous [80], with only limited diatom production occurring in the pond itself. The assemblage shift ~1880 AD, which continues after the 1980s, is similar to the Condition 1 to Condition 2 transitions of the other “cool” sites, with a decrease in epipelic *Amphora* taxa and increases in nutrient-associated *Nitzschia* spp. (*N*. *alpina*, *N*. *frustulum*, and *N*. *perminuta*), and eurytopic *Achnanthidium minutissimum*.

The initial assemblages differed between the “cool” sites (Fig 5) and the “warm” sites (Fig 4), the latter being dominated by benthic fragilarioid taxa, and the former having a higher initial diversity than their “warm” counterparts. This is likely due to differences in their local conditions, particularly catchment morphology and geology. The benthic fragilarioid taxa are alkaliphilic [65]. For Paradise Pond and Plateau Pond 2, the lack of benthic fragilarioid taxa may be due to their lower buffering capacity as, due to their relatively higher elevations, they have little calcareous till in their catchments, which is also reflected in the water chemistry (Table 2). Paradise Pond in particular has some of the lowest pH measurements of the Cape Herschel ponds. Although the pH values indicated slightly alkaline conditions at the height of summer in July 2011, prior sampling events measured pH in the mid-low 6s. Rather than a dominance of benthic fragilarioids, a collection of acidophilic (e.g. *Diadesmis gallica* [51]) and epipelic (e.g. *Eucocconeis leptostriata*, *Amphora inariensis*) species characterizes the pre-shift assemblages, assemblages that are typical for poorly buffered Arctic sites [69, 81, 82]}. While the “cold” lakes have similar DIC concentrations to the poorly-buffered “cool” sites, these deeper lakes have had, until recently, persistent ice pans in summer. Benthic fragilarioids likely dominate the records from these “cold” lakes as these taxa do well under low light conditions [65, 68].

Moraine Pond does have calcareous till in its catchment, however it is fed by a stream and drains a much larger area than the other Cape Herschel ponds (including the large Cape Herschel cliffs that are now actively thawing). Consequently, the pond is mesotrophic (Table 2), and *Nitzschia* spp. (*N*. *alpina*, *N*. *frustulum*, and *N*. *perminuta*), common Arctic taxa that dominate assemblages at sites with greatly elevated nutrients [73, 74] and more alkaline pH [78, 83], are present in high relative abundances throughout the Moraine Pond record.

### Changes in primary production trends

The chl *a* profiles (Fig 8E–8H) record increases in primary production relative to their background values in nine sites, which were generally in good agreement with the diatom assemblage changes at many of the sites, as reflected by the changing CONISS zonations. Sedimentary chl *a* has been used successfully to track production-related changes associated with climate warming in a number of Arctic lakes [52, 53, 84]. Although the proportion of inorganic inputs can dilute a production signal, with warmer conditions and longer growing seasons, we hypothesize an increase in overall aquatic production, a trend that has been documented across a number of Arctic regions [84, 85]. While the changes in diatom assemblages and primary production may be partially explained by increased nutrients as the result of the “greening” of the catchments with warming [76, 77], we would expect that waterbodies with similar nutrient concentrations (higher nutrient sites: Moraine Pond and the “oasis” sites, versus lower nutrient sites: Col Pond, Elison Lake, High Lake, Proteus Lake, and West Lake) would have similar responses, and that is not the case at our sites.

Given our hypothesis that the ice-free period is the main driver of overall limnological changes, we would expect that, in sites where ice cover remains persistent, there would be little change in primary production. Two of our “cold” sites, High Lake and Proteus Lake, record profiles that are consistent with this hypothesis, as production remains stable until the most recent intervals (Fig 8G). The chl *a* increases at these two sites are concurrent with the emergence of the epiphyte *Hygropetra balfouriana*, albeit at low abundances, in the records (Fig 6A and 6B). The algal changes in the upper intervals of these sites reflect the striking changes in ice cover reduction that we have observed in recent years. For example, in 2011, Pim Island sites recorded, for the first time in our 30 years of sampling, less than 90% July ice coverage. The chl *a* concentrations in the West Lake sediment core increase after ~1850 AD, after which production is relatively high, but variable (Fig 8G). The increase in chl *a* perhaps reflects the onset of a longer open water period that has not yet undergone a substantial enough change to elicit the development of new diatom habitats, as the change in primary production does not appear to be related to either the diatom diversity (Fig 8C) or individual species changes (Fig 6C). Of course, the increased production may also be linked to changes in other algal groups.

Primary production increased in the “warm” group (Fig 8E) and the “cool” group (Fig 8F), coincident with the diatom assemblage shifts, but with the changes occurring later at Moraine Pond and Paradise Pond as compared to the changes occurring around 1850 AD at Col Pond. Again, this is consistent with our hypothesis, where local conditions are resulting in variation in the ice-free period, leading to lags in the development of aquatic vegetation in shaded or high elevation sites, compared to their lowland counterparts.

While the “oasis” sites were not anticipated to show marked changes in diatom species diversity (Fig 8D), it is likely that the ice-free period would still have been extended in response to warming. As expected, both “oasis” sites record increased primary production (Fig 8H). At SV Pond 8, the increase occurs just after the peak of the industrial period (~1870 AD), highlighting the sensitivity of the Arctic to even small changes in climate [4]. Similarly, at SV Pond 5, the increase in production is coincident with changes in the diatom assemblage (Fig 7A); however, we cannot pinpoint a date for these changes due to the low ^210^Pb activities at this site.

### Mechanisms of change

The transition in the sediment record from Condition 1 to Condition 2 is a consistent trend recorded in the “warm”, “cool”, and, to an extent, “cold” sites. For our Arctic ponds, whose entire water columns completely freeze during winter, the length of the ice-free season is likely the primary determinant of aquatic moss establishment and, consequently, the proliferation of epiphytic diatom species. Aquatic moss growth at high latitudes is largely determined by underwater irradiance, which is affected by the length of the day, sun’s elevation, and the snow and ice cover [86]. In a study on aquatic moss in a High Arctic lake, 90% of moss growth was attributable to the amount of snow cover and July irradiance [86]. Additionally, increased nutrient inputs, due to warmer water temperatures in response to climate change and higher weathering rates, may also encourage moss proliferation [87].

The largely synchronous increases between primary production and the establishment of more complex epiphytic diatom assemblages in our study might also, in part, be a reflection of the suitability of the new moss habitat for diatom colonization. It has been suggested that vegetation substrates may release carbohydrates and nutrients into the water to be taken up by epiphytic diatoms [88, 89], potentially promoting growth and may partially explain the coincident timing of inferred production and epiphytes. The variability of the vegetative substrate, the additional competitive advantage it affords, and the lengthened growing season, which allows for greater complexity [90], may contribute to the increased diversity in epiphytic diatom communities in contrast to their epilithic and epipelic counterparts.

### Context is key

We postulated that the recent warming trend in the Canadian High Arctic is increasing the ice-free period of lakes and ponds, but that this ice-free period is also partly controlled by local conditions. The four site categories exhibited different timings and degrees of diatom species change that we suggest are based on site-specific and localized differences in catchment characteristics and setting that affected local ice cover dynamics. Two of the groups (the “warm” sites and the “cool” sites) are located in the polar desert and presently have relatively long ice-free periods, which, due to regional warming, are expected to have experienced a lengthening of the ice-free period over the Anthropocene. The “warm” sites and the “cool” sites recorded anticipated switches in diatom assemblage composition, from epilithic and epipelic assemblages, to more diverse and productive states with the development of diatoms with complex growth forms and the establishment of epiphytic diatom species. The “cold” sites have yet to undergo, or have recently begun undergoing, this shift in conditions, as substantial ice cover persist at these sites even as the region, as a whole, has warmed since the mid-19th century. In contrast to the “warm” and “cool” sites, the historically warm “oasis” sites recorded only subtle diatom species changes, as was expected, but continued warming, together with a further extension of the growing season over recent decades, resulted in a marked increase in chl *a*. Although primary production for our sites appears to be, at least partially, directly related to increases in the ice-free period due to warming temperatures, the diatom compositional shifts are largely indirectly related to the ice-free season and are rather responding to changes in habitat associated with longer growing seasons.

There has been marked turnover in diatom taxa in lakes and ponds across the Arctic in response to climate change [5–7]. Our comparative paleolimnological approaches show that increases in primary production and shifts in diatom assemblages are responding indirectly to regional climate warming and, as hypothesized, can be shown to be further moderated by local ice cover dynamics and vegetative habitat availability.

## Supporting information

S1 Table Field note observations of ice-cover and snow. Field note observations of ice-cover and snow near the lake or pond for the 10 study sites (visited intermittently from 1983–2011), supporting the division into “warm”, “cool”, “cold”, and “oasis” sites.
(DOCX)
