# Supplementary material for: Ice-cover is the principal driver of ecological change in High Arctic lakes and ponds
Source: PLoS One. 2017 Mar 15;12(3):e0172989. doi: 10.1371/journal.pone.0172989 (PMC5351862; doi:10.1371/journal.pone.0172989)
Supplement: S1 Table — Field note observations of ice-cover and snow near the lake or pond for the 10 study sites (visited intermittently from 1983–2011), supporting the division into “warm”, “cool”, “cold”, and “oasis” sites. (DOCX) [file pone.0172989.s001.docx]

| Site Group | Lake Name | Elevation (m a.s.l.) | Date sampled | Estimated % Ice cover | Snow adjacent to waterbody (Y/N) | Comments |
| --- | --- | --- | --- | --- | --- | --- |
| "Warm" Site | Col Pond  (78° 36.154' N,  74° 39.758' W) | 137 | June 19 1983 | 0 | Y |  |
|  |  |  | June 11 1984 | 0 | Y | First pond on the Cape to thaw completely |
|  |  |  | June 21 1986 | 5 | Y | The 1986 field season on Cape Herschel extended from June-August. Col Pond was almost entirely open June 21, 1986 |
|  |  |  | June 23 1986 | 0 | N | Ice-free |
|  |  |  | June 26 1986 | 0 | N | Ice-free |
|  |  |  | June 30 1986 | 0 | N | Ice-free |
|  |  |  | July 6 1986 | 0 | N | Ice-free |
|  |  |  | July 13 1986 | 0 | N | Ice-free |
|  |  |  | July 24 1986 | 0 | N | Ice-free |
|  |  |  | July 29 1986 | 0 | N | Ice-free |
|  |  |  | August 6 1986 | 100 | Y | Ice and slush covered |
|  |  |  | August 14 1987 | 0 | N |  |
|  |  |  | August 24 1987 | 30 | Y | Covered by 1 cm ice; re-visited on Aug 27 and pond was still ice-free in the center, but now had 3 cm thick ice in the shallow regions. |
|  |  |  | July 15 1995 | 0 | N |  |
|  |  |  | July 14 1998 | 0 | N |  |
|  |  |  | July 15 2001 | 0 | N |  |
|  |  |  | July 17 2004 | 0 | N |  |
|  |  |  | July 11 2006 | 0 | N |  |
|  |  |  | July 9 2007 | 0 | N |  |
|  |  |  | July 14 2009 | 0 | N |  |
|  |  |  | July 18 2011 | 0 | N |  |

| Site Group | Lake Name | Elevation (m a.s.l.) | Date sampled | Estimated % Ice cover | Snow adjacent to waterbody (Y/N) | Comments |
| --- | --- | --- | --- | --- | --- | --- |
| "Warm" Site | Elison Lake  (78° 36.487' N,  74° 44.414' W) | 23 | June 28 1983 | 30 | Y |  |
|  |  |  | June 12 1984 | 90 | Y | Field notes indicate it lost full ice cover by June 30, 1984 |
|  |  |  | June 19 1986 | 100 | Y | Still frozen |
|  |  |  | June 24 1986 | 95 | Y | Just began to open up |
|  |  |  | June 29 1986 | 95 | Y | Only a moat of open water |
|  |  |  | July 5 1986 | 85 | Y |  |
|  |  |  | July 13 1986 | 55 | Y |  |
|  |  |  | July 22 1986 | 0 | N | Ice-free |
|  |  |  | August 3 1986 | 0 | N | Ice-free |
|  |  |  | August 17 1987 | 0 | N |  |
|  |  |  | August 25 1987 | 50 | Y | South end is completely ice free, north end has developed 1.2 cm ice |
|  |  |  | August 28 1987 | 50 | Y | Some ice, but still some open water. Elison Lake is the last on the Cape to develop full ice |
|  |  |  | July 17 1995 | 0 | Y |  |
|  |  |  | July 9 1998 | 0 | N |  |
|  |  |  | July 13 2001 | 0 | N |  |
|  |  |  | July 22 2004 | 0 | N |  |
|  |  |  | July 13 2006 | 0 | N |  |
|  |  |  | July 10 2007 | 0 | N |  |
|  |  |  | July 14 2009 | 0 | N |  |
|  |  |  | July 16 2011 | 0 | N |  |
|  |  |  |  |  |  |  |

| Site Group | Lake Name | Elevation (m a.s.l.) | Date sampled | Estimated % Ice cover | Snow adjacent to waterbody (Y/N) | Comments |
| --- | --- | --- | --- | --- | --- | --- |
| "Cool" Site | Moraine Pond  (78° 36.685' N,  74° 40.977' W) | 89 | June 26 1983 | 80 | Y |  |
|  |  |  | July 1 1983 | 80 | Y |  |
|  |  |  | June 30 1986 | 100 | Y | Still under snow and ice |
|  |  |  | July 8 1986 | 90 | Y | Majority of the pond still under snow and ice |
|  |  |  | July 20 1986 | 100 | Y | Still covered by snow drift |
|  |  |  | August 15 1987 | 0 | Y |  |
|  |  |  | August 24 1987 | 100 | Y | Covered by 1.6 cm ice |
|  |  |  | August 27 1987 | 100 | Y | Covered by 2.3 cm ice and snow |
|  |  |  | July 13 1995 | 0 | Y | Large snowbank on cliff side |
|  |  |  | July 14 1998 | 0 | Y |  |
|  |  |  | July 10 2001 | 0 | Y |  |
|  |  |  | July 17 2004 | 90 | Y |  |
|  |  |  | July 11 2006 | 0 | Y |  |
|  |  |  | July 21 2007 | 50 | Y |  |
|  |  |  | July 14 2009 | 50 | Y |  |
|  |  |  | July 18 2011 | 0 | Y | Very thin snowbank that completely disappeared by July 20, 2011 (for the first time ever in our 32 years of site visits) |

| Site Group | Lake Name | Elevation (m a.s.l.) | Date sampled | Estimated % Ice cover | Snow adjacent to waterbody (Y/N) | Comments |
| --- | --- | --- | --- | --- | --- | --- |
| "Cool" Site | Paradise Pond  (78° 36.530' N,  74° 46.117' W) | 134 | July 5 1986 | 0 | Y | Large snowbank on north edge |
|  |  |  | July 22 1986 | 0 | Y | Snow persistent on north edge |
|  |  |  | August 22 1987 | 0 | N |  |
|  |  |  | July 17 1995 | 0 | Y |  |
|  |  |  | July 9 1998 | 0 | N |  |
|  |  |  | July 13 2001 | 0 | Y |  |
|  |  |  | July 20 2004 | 0 | N |  |
|  |  |  | July 13 2007 | 0 | Y |  |
|  |  |  | July 13 2007 | 0 | N |  |
|  |  |  | July 15 2009 | 0 | Y |  |
|  |  |  | July 16 2011 | 0 | Y |  |
| "Cool" Site | Plateau Pond 2  (78° 35.500' N, 74° 38.427' W) | 246 | July 20 1983 | 0 | Y |  |
|  |  |  | June 17 1984 | 10 | Y |  |
|  |  |  | June 21 1986 | 100 | Y | Totally ice-covered with 30 cm of snow on top |
|  |  |  | July 6 1986 | 80 | Y | Mostly ice-covered |
|  |  |  | August 6 1986 | 100 | Y | Thick ice cover and slush to the bottom of pond |
|  |  |  | August 24 1987 | 100 | Y | All frozen slush, 2.5 cm ice |
|  |  |  | August 27 1987 | 100 | Y | Covered by 4.2 cm ice |
|  |  |  | July 12 1995 | 0 | Y |  |
|  |  |  | July 14 1998 | 50 | Y |  |
|  |  |  | July 10 2001 | 30 | Y |  |
|  |  |  | July 17 2004 | 0 | Y |  |
|  |  |  | July 11 2006 | 0 | Y |  |
|  |  |  | July 9 2007 | 2 | Y |  |
|  |  |  | July 14 2009 | 0 | Y |  |
|  |  |  | July 18 2011 | 0 | Y |  |

| Site Group | Lake Name | Elevation (m a.s.l.) | Date sampled | Estimated % Ice cover | Snow adjacent to waterbody (Y/N) | Comments |
| --- | --- | --- | --- | --- | --- | --- |
| "Cold" Site | High Lake  (78° 42.700' N,  74° 22.283' W) | 463 | July 13 2011 | 0 | Y | Rocky, un-vegetated catchment. This site has previously not been visited as it was not accessible due to extensive snow and ice cover |
| "Cold" Site | Proteus Lake (78° 41.876' N,  74° 23.022' W) | 376 | July 19 1983 | 80 | Y |  |
|  |  |  | August 19 1987 | 80 | Y |  |
|  |  |  | July 15 1998 | 95 | Y |  |
|  |  |  | July 24 2007 | 30 | Y |  |
|  |  |  | July 11 2009 | 60 | Y |  |
|  |  |  | July 13 2011 | 20 | Y |  |
| "Cold" Site | West Lake  (78° 44.491' N,  74° 37.751' W) | 323 | June 23 1983 | 100 | Y |  |
|  |  |  | August 19 1987 | 40 | Y |  |
|  |  |  | July 11 2009 | 90 | Y |  |
|  |  |  | July 14 2011 | 0 | Y |  |
|  |  |  |  |  |  |  |
| Site Group | Lake Name | Elevation (m a.s.l.) | Date sampled | Estimated % Ice cover | Snow adjacent to waterbody (Y/N) | Comments |
| "Oasis" Site | Sverdrup Pond 5 (79° 7.951' N,  79° 48.582' W) | 299 | July 12 2011 | 0 | N | 100% vegetated shoreline |
| "Oasis" Site | Sverdrup Pond 8 (79° 7.680' N,  79° 58.498' W) | 296 | July 4 1984 | 0 | N | Photo of SV Pond 8 shows abundant vegetation surrounding the pond perimeter |
|  |  |  | July 12 2011 | 0 | N | 100% vegetated shoreline |
